# Inferior vena cava leiomyosarcoma: vascular reconstruction is not always mandatory

**DOI:** 10.11604/pamj.2016.24.287.8912

**Published:** 2016-07-29

**Authors:** Maher Slimane, Nada Belhaj Yahia, Hanene Bouaziz, Hatem Bouzaine, Jamel Benhassouna, Tarek Ben Dhieb, Monia Hechiche, Amor Gammoudi, Khaled Rahal

**Affiliations:** 1 Oncologic Surgery Department, Institut Salah Azaiez, Tunis, Tunisie; 2 Anatomical Pathology Department, Institut salah Azaiez, Tunis, Tunisie

**Keywords:** Leiomyosarcoma, vena cave, reconstruction, prosthesis

## Abstract

Leiomyosarcoma (LMS) of inferior vena cava is a rare and aggressive tumor, arising from the smooth muscle cells in the vessel wall. A large complete surgical resection is the essential treatment. The need of vascular reconstruction is not always mandatory. It’s above all to understand the place of the reconstruction with artificial vascular patch prosthetics of vena cave after a large resection of the tumor. We rapport two cases of LMS of inferior vena cava in two women who underwent successful large resection of tumor and lower segment of inferior vena cava. In first case, reconstruction of the inferior vena cava was not performed because of the development of venous collaterals derivation. In the second case reconstruction was done using Dacron interposition graft. The necessity of a large resection in management of primary leiomyosarcoma of vena cave makes sometimes unavoidable the sacrifice of a portion of the vena. Indeed, a better comprehension of the development of venous derivation may render unnecessary the reconstruction.

## Introduction

Leiomyosarcoma (LMS) of the inferior vena cava (IVC) are rare tumors with only a hundred cases reported in the literature. These tumors are characterized by slow growth, late diagnosis and poor prognosis. Only one piece resection of the tumor carrying the segment reaches the inferior vena cava will allow a better survival. In some cases vascular reconstruction of the inferior vena cava is not necessary considering the development of a collateral circulation.

## Methods

Were included in our study, 2 cases of leiomyosarcoma of the inferior vena cava, deemed operable.

## Results

### Case 1

**Figure 1:**
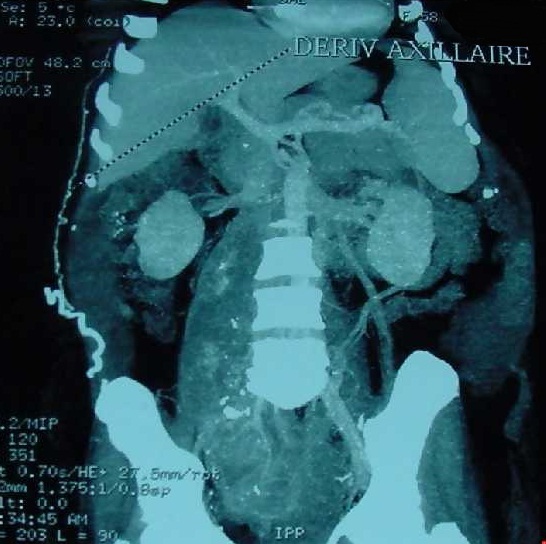
Abdominal CT angiography: axillary vein bypass

**Figure 2:**
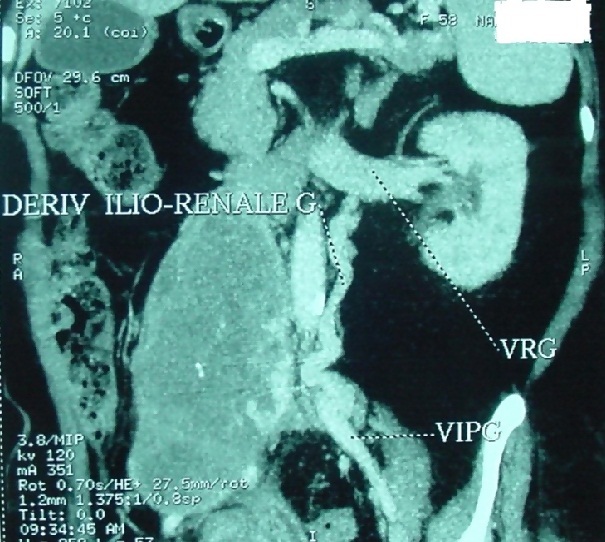
Abdominal CT angiography: left ilio-renal shunt

**Figure 3:**
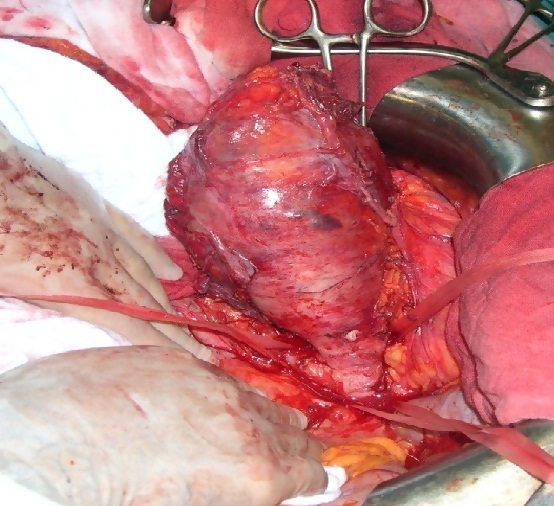
Operative view: tumor of the inferior vena cava

A 58 year old woman, with no history, who has presented for a year pelvic pain associated with pelvic heaviness sensation and dysuria. Clinical examination found a bloated abdomen, with perception of a hard mass, reduced mobility, right latero-rectal rectal examination and no lower limb edema. Abdominal CT scan showed an abdominal pelvic tissue mass 200 × 180 × 160 mm with IVC thrombus tissue and right common iliac vein. The CT angiography objectified a large heterogeneous tissue process partially calcified IVC infrarenal fusiform 235 × 75 mm with endo -pelvic right extension and highlighted venous shunts. These diversions were walking from the right common femoral vein into the thoracic base and to the right axillary hollow (Figure 1). The right external iliac vein was totally collapsed. The right common iliac vein, taken in the mass, was not visible. There was also an iliac branch left renal vein by left para-lumbar veins and a big left renal vein (Figure 2). Chest CT scan showed no pleuropulmonary metastasis. The biopsy under scanner concluded an LMS low malignancy. During surgery, there is a tumor of the inferior vena cava sub renal 250 × 90 mm, coming in contact with the bifurcation of the common iliac veins. Exploring the rest of the peritoneal cavity found neither impaired hepatic function nor carcinomatosis. We proceeded to a dissection of the infrarenal IVC and individualized the renal veins that have been placed into vessel loops. Then ligation was carried out and the section of the iliac veins and right gluteal vein to their origin and sectioned VCI at the lower edge of the right renal vein to allow removal of the mass of the IVC and iliac veins (Figure 3).

### Case 2

**Figure 4:**
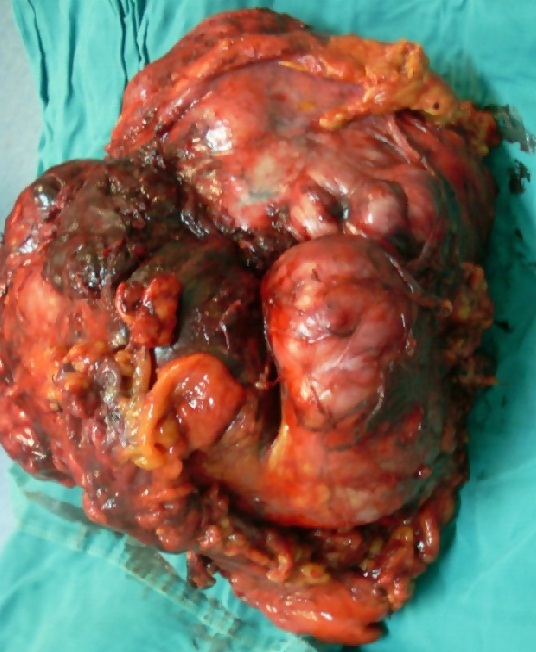
Resection of the mass carrying the piece and invaded part of the inferior vena cava

53 year old woman, with no particular history, had had for 6 months abdominal pain in a context of impaired general condition. Abdominal examination found a painless mass of mobile right flank, 150 x100 mm major axis. In a vaginal touch is palpated a right adnexal mass, mobile and enclosed in the pouch of Douglas. Abdominal ultrasound showed a right para umbilical mass, which was 110 x 120 mm long axis of undetermined origin with right ureterophrosis. The thoraco-abdominal-pelvic CT scan found a mass of intraperitoneal right, oval, with lobed edges, heterogeneous, with foci of necrosis, measuring 120x80mm. It delivers the cecum and small bowel loops side forward and inward. Iliac arteries and veins were in contact with the ground but without signs of invasion. The lower vena cava on kidney was laminated to the iliac bifurcation but remained permeable. The tumor was pressing and included the right ureter to the height of L1 to L3, with dilatation of the right ureteral and pyelocalyceal cavities. Chest CT scan showed no pulmonary side locations. Pathological examination on scan guided biopsy specimen has concluded to sarcoma. We performed resection of the mas, carrying the piece in the right kidney and the basement flooded portion of the inferior vena and a bifurcated Dacron prosthesis was asked to ensure venous reconstruction (Figure 4). Pathological examination and the immuno histochemical profile positive for anti-vimentin antibody and anti-actin were consistent with the diagnosis of leiomyosarcoma. The postoperative course was uneventful. A radiation dose of 45 Gy was made for a better local control.

## Discussion

The tumors of the IVC are rare. They represent 0.07% case of an autopsy series [1]. Of these tumors, 95% are LMS [2]. There are 250 cases of leiomyosarcoma of inferior vena cava reported worldwide. The vascular leiomyosarcoma are malignant mesodermal tumors developed from the smooth muscle fibers of the media [3]. It affects 80% of women [4], with an average age of 58 at diagnosis [5]. From a surgical point of view we divide the VCI in 3 portions [6]: Segment I, infrarenal, the lower segment, it is affected in 28% of cases; Segment II that extends from the origin of the renal veins to the origin of the hepatic veins, the middle segment. It represents the most affected segment with a frequency of 55%; Segment III ranging from suspension hepatic veins to the right atrium, the upper segment and it is the least affected with a frequency of 17%.

The LMS endoluminal development mainly concern retrohepatic region tumors and a complete occlusion of the vascular lumen may complicate the course. However the exoluminal development interest especially liver tumors portion. The deep location of LMS explains nonspecific and late symptoms of these tumors [5]. Indeed, clinical signs depend primarily on the location of the tumor and its size: the concept of pain of hypochondria is often present whatever the localization of the tumor; for the tumor portion in liver, palpation of a mass in the review is a circumstance of quite frequent discovery; venous signs: lower limb edema, subcutaneous collateral circulation, thrombophlebitis can be observed but are quite rare because of the progressive development of the tumor leaving time to develop a substitute system. Tumors reaching the hepatic vein may even give a Budd-Chiari syndrome [6, 7]. The thoraco-abdominal-pelvic CT is the reference examination for asking diagnosis, it typically shows a poly lobed mass with inhomogeneous contrast enhancement. It also enables the evaluation of loco regional extension, it specifies the degree of obstruction of the IVC and it enables search for metastases. The magnetic resonance imaging is even more powerful than the scanner to identify the vascular reports. A sonographically or scannographically guided biopsy allows a histological evidence before surgery [6]. The wide excision is the mainstay of the treatment of leiomyosarcoma of the IVC and is often associated with the resection of the IVC [7]. The reconstruction of the IVC has a limited role. The prosthetic replacement with prosthesis TPFE represents a significant risk of infection and thromboembolism. Indeed, a resection of the IVC without venous reconstruction in a patient with a well-developed network locum can be well tolerated. The complexity of the surgical procedure depends essentially on the location of the tumor [8]: tumors of segment I are the easiest to treat; tumors of segment II are the most common laying the conservation problem of the right renal vein; tumors of segments III are considered inoperable exceptions, or the tumor is completely resected with venous reconstruction gesture prosthesis and associated with liver transplantation. Preoperative radiotherapy is indicated to reduce tumor size in postoperative it appears to reduce the rate of recidivism and improve local control [9]. Chemotherapy has not been proven effective [8], though it remains indicated for inoperable tumors to reduce the size LMS high grade, metastatic immediately the sick, the rapid recurrence and in young subjects. However, it is noted that the rarity of the disease explains why there are no studies to judge the effectiveness of different treatments. The trend is especially marked by the risk of recurrence, exceeding 55%, and by the appearance of distant metastases [10]. Indeed survival at 5 years was only 31% [11].

## Conclusion

The leimyosarcomes of IVC are rare tumors. Because of their non-specific symptoms and their clinical latenc, their support is often late and the prognosis is unfortunate. The contribution of medical imaging allowed having an accurate assessment of the lesions and mapping the Contingency network to predict surgical difficulties. The treatment is based on an oncologic resection associated with radio-chemotherapy. But still frequent recurrence despite optimal treatment and regular monitoring is required.

### What is known about this topic

- Leiomyosarcoma of the inferior vena cava are rare and aggressive tumors;
- Surgical management is based on a one piece resection of the tumor carrying the segment reaches the inferior vena cava;
- Even if the treatment is based on the surgery associated with a radio-chemotherapy, the support arrangements vary from case to case.

### What this study adds

- Although vascular resection is necessary in management of inferior vena cava leiomyosarcoma, vascular reconstruction is not always mandatory;
- The contribution of medical imaging allowed having an accurate assessment of the lesions and mapping the contingency network to predict surgical difficulties;
- Sacrificing inferior vena cava not associated with more morbidity in our case because of the importance of the collateral circulation.
